# The role of socio-economic status in depression: results from the COURAGE (aging survey in Europe)

**DOI:** 10.1186/s12889-016-3638-0

**Published:** 2016-10-19

**Authors:** Aislinne Freeman, Stefanos Tyrovolas, Ai Koyanagi, Somnath Chatterji, Matilde Leonardi, Jose Luis Ayuso-Mateos, Beata Tobiasz-Adamczyk, Seppo Koskinen, Christine Rummel-Kluge, Josep Maria Haro

**Affiliations:** 1 Klinik und Poliklinik für Psychiatrie und Psychotherapie der Universität Leipzig, Leipzig, Germany; 2 Parc Sanitari Sant Joan de Déu, Universitat de Barcelona, Fundació Sant Joan de Déu/CIBERSAM, Dr. Antoni Pujadas, 42, Sant Boi de Llobregat, 08830 Barcelona, Spain; 3 Department of Health Statistics and Information Systems, World Health Organization, Geneva, Switzerland; 4 Department of Neurology, Public Health and Disability, Italian National Neurological Institute “Carlo Besta” Foundation IRCCS (Istituto di ricovero e curaa carattere scientifico), Milan, Italy; 5 Instituto de Salud Carlos III, Centro de Investigación Biomédica en Red de Salud Mental, CIBERSAM, Monforte de Lemos 3-5, Pabellón 11, 28029 Madrid, Spain; 6 Department of Psychiatry, Universidad Autónoma de Madrid, Instituto de Investigación Sanitaria Princesa (IP), Hospital Universitario la Princesa, Madrid, Spain; 7 Epidemiology and Preventive Medicine, Department of Medical Sociology, Jagiellonian University Medical College, Krakow, Poland; 8 National Institute for Health and Welfare, Helsinki, Finland; 9 Parc Sanitari Sant Joan de Déu, Fundació Sant Joan de Déu, CIBERSAM, Dr. Antoni Pujadas, 42, 08830 Sant Boi de Llobregat, Barcelona, Spain

**Keywords:** Depression, Socioeconomic status, Cross-national, Income, Education

## Abstract

**Background:**

Low socio-economic status (SES) has been found to be associated with a higher prevalence of depression. However, studies that have investigated this association have been limited in their national scope, have analyzed different components of SES separately, and have not used standardized definitions or measurements across populations. The aim of the current study was to evaluate the association between SES and depression across three European countries that represent different regions across Europe, using standardized procedures and measurements and a composite score for SES.

**Method:**

Nationally-representative data on 10,800 individuals aged ≥18 from the Collaborative Research on Ageing in Europe (COURAGE) survey conducted in Finland, Poland and Spain were analyzed in this cross-sectional study. An adapted version of the Composite International Diagnostic Interview was used to identify the presence of depression, and SES was computed by using the combined scores of the total number of years educated (0–22) and the quintiles of the country-specific income level of the household (1–5). Multivariable logistic regression was used to assess the association between SES and depression.

**Results:**

Findings reveal a significant association between depression and SES across all countries (*p* ≤ 0.001). After adjusting for confounders, the odds of depression were significantly decreased for every unit increase in the SES index for Finland, Poland and Spain. Additionally, higher education significantly decreased the odds for depression in each country, but income did not.

**Conclusion:**

The SES index seems to predict depression symptomatology across European countries. Taking SES into account may be an important factor in the development of depression prevention strategies across Europe.

## Background

Depression is a significant public health issue which transcends communities and countries. It is the leading cause of disability worldwide, and the global burden of depression is on the rise [1, 2]. The prevalence of depression varies considerably both within and between countries across Europe [3, 4], which may be a reflection of the role of contextual factors, such as economic, demographic and environmental factors on the development and prevalence of depression [5–9]. Beset by growing national and international inequalities in income, education and wealth, socioeconomic status (SES) has come into focus as a crucial determinant of depression [10]. The role of SES in depression is an important theme, and there is a large body of literature which illustrates the negative association between SES and depression [11–13] where according to Lorant and colleagues, low SES-individuals have higher odds of being depressed [14].

To date, many studies have evaluated the role of SES on depression, using individual levels of stratification related to income, education, occupation, social class, or wealth [10, 13–17]. However, despite this, there is a dearth of research that uses standardized measures or definitions for SES, or that compare this association between countries of different socio-economic and cultural contexts using such measures. The Collaborative Research on Ageing in Europe (COURAGE), from which our data was derived, is among the few large population-based nationally-representative health studies that apply standardized designs and procedures across all survey populations. The countries included in the COURAGE survey were deliberately chosen to represent different cultural and economic statuses in Europe.

As yet, there has been no “gold standard” put forward for measuring SES, and as such, due to variation in measurement techniques, the transferability and comparability of existing findings are limited. Traditionally, the measurement of SES can encompass several different indicators, which often results in gradients of varying slopes [18]. Moreover, the numerous interchangeable terms used to describe SES create complexity in interpreting findings. Using standardized measurements and definitions for SES allows for comparison, particularly between different countries, and ensures that the same component is being measured. This is important particularly for good research practice, and for clinical application.

In addition to this, the majority of the studies investigating this association are not representative of an entire population or country, nor have previous studies focused collectively on the current countries in question. Moreover, in epidemiological studies investigating the relationship between depression and measures of SES, standardized measurements or definitions for SES and depression were lacking.

Given the increasing burden of depression globally [6], the deepening challenge of income inequality [19], as well as the lack of global evidence on the association between depression and SES among cross-country populations using systematic measures, the aim of the present study was to evaluate and compare the association between SES (as a composite score), education and income with depression in three model European countries.

## Methods

### Design

The COURAGE was a cross-sectional, general population survey of non-institutionalized adults (aged ≥18 years), conducted in Finland, Poland and Spain between 2011 and 2012 (*n* = 10,800 individuals: Finland 1976; Poland 4071; and Spain 4753). These countries were selected to give a broad representation across different European regions, representing the north, the east and the south of Europe respectively, and taking into consideration their populations, health and welfare characteristics (median age, life expectancy and sex ratio) [20]. In Poland and Spain, a stratified multistage clustered design was used using strata according to geographical administrative and catchment area sizes. Municipalities and census units were systematically selected with probabilities proportional to the population size. Age strata were used to select households, and individuals were randomly selected from inhabitants in a certain age group within the household. In Finland, a two-stage clustered sampling design was used and strata were created based on the largest towns and university hospital regions. Systematic sampling was conducted so that the sample size in each stratum was proportional to the base population. The differences in socio-economic gradients between Finland, Poland, and Spain provide opportunities to compare the effects of social security mechanisms and aging outcomes. The individual response rates were 53.4, 66.5 and 69.9 %, in Finland, Poland and Spain respectively. Sampling weights were generated to account for the complex study design in each country. Post-stratification corrections were made to the weights to adjust for the population distribution obtained from the national census from each country.

Information was gathered through household interviews, where interviews were conducted face-to-face by Computer-Assisted Personal Interviewing (CAPI). All the interviewers participated in a training course for the administration of the survey. Quality control procedures were implemented during fieldwork [17]. The instruments were translated from English into Finnish, Polish, and Spanish following the World Health Organization (WHO) translation guidelines for assessment instruments, which included a forward translation, a targeted back-translation, review by a bilingual expert group and a detailed translation report [21]. These three countries followed this same systematic methodology and utilized the same standardized questionnaire to collect information on health and well-being among non-institutionalized adult populations. Further details of the survey can be found elsewhere [22].

### Depression

An adapted version of the Composite International Diagnostic Interview (CIDI 3.0) was used to assess the presence of depression in the previous 12 months [23]. Depression was confirmed if a certain number of symptoms of depression were endorsed by the respondent, as calculated by an algorithm based on the DSM-IV for Major Depressive Disorder [24].

### Socio-demographic and lifestyle characteristics

Participants were asked to provide various socio-demographic information (e.g. age and sex). Marital status was categorized as single/never married; married/co-habiting; or divorced/widowed. Country-wise quintiles of household income before tax were calculated asking the specific question ¨ which categorization best represents the total personal earnings income of all family members (including yourself) in the past 12 months, before taxes? (Count only wages and other stipends from their employment, not pensions, investments, or other income)¨. Next the household income variable was corrected with the use of household income by social security retirement benefits, by any income from government assistance programs and other sources (i.e., pensions, investments, child support or alimony). The 1st quintile and the 5th quintile represented the lowest and the highest level of wealth respectively. Education was based on the total number of years of education received, with 0 being the minimum and 22 the maximum.

### Socio-economic status

The indicators education and income have been chosen as components of the SES index as they have strong theoretical associations with depression [18, 25–30]. The use of years educated and income level as components of the SES index is also supported as they are more applicable to modern society, are based mainly on numerical, self-report data, and are easy to obtain. The omission of occupation based measures can be justified because it is not applicable to people who are currently unemployed (students, jobless individuals, retired people, stay-at-home mothers, etc.), many occupational based measures are outdated [31] and also because occupation may have different meanings for different birth cohorts and in different geographical settings (which may make international comparisons problematic) [18]. Based on existing literature [32–36], a composite score for SES, using the determinants of education and income, was generated. Education and income included in the same model may lead to biased estimates due to collinearity, therefore, the composite score was generated in order to counteract this. The composite score of SES was computed by using the total number of years educated (0–22) and the quintiles of the country-specific income level of the household (1–5). Education level and household income level in multi-adjusted models could also be independently added. However, this analysis would raise co-linearity issues, which influences the robustness of the model’s estimates. In order to provide an accurate estimate of respondents’ SES, these two variables were multiplied to create combined scores ranging from 0 to 110.

### Statistical analysis

Data was available on 10,800 participants (Finland, 18 %; Poland, 38 %; Spain 44 %). Country-wise analyses were conducted to account for the heterogeneity between countries. The baseline characteristics were compared between depressed and non-depressed. Continuous variables (SES index) was presented as mean ± SD, and categorical variables as percentages. Chi square tests were used to test the association between depressed and non-depressed for each variable. Logistic regression analyses with multiple variables were conducted to assess the association between education, income or SES index (independent variable) and depression (dependent variable) for the individual countries. All models were adjusted for age, sex, and marital status. The results illustrating education and income included in the models individually have been presented in order to enhance our knowledge of those aspects of SES that are critical for depression, and also to facilitate comparisons with existing studies. The sample weighting and the complex study design were taken into account in all analyses to generate nationally-representative estimates. Results from regression models are presented as odds ratios and 95 % Confidence Intervals (CIs). All reported *p*-values were based on two-sided test, where the level of statistical significance was set at *p* < 0.05. SPSS software, version 19 was used for all statistical calculations (SPSS Inc., Chicago IL, USA).

## Results

The analytical sample size was 10,800 (Finland, 1976; Poland, 4071; Spain, 4753). The mean age ± SD in Finland was 50 ± 0.43, 46 ± 0.42 in Poland, and 48 ± 0.33 in Spain. The prevalence of depression was 4 % in Finland and Poland, and 9 % in Spain (*p* ≤ 0.001), where females had a significantly higher prevalence of depression than males in each country (≤0.001). In terms of age groups, Finland had a higher proportion of the younger age group who were categorized as depressed, and Spain had a higher proportion of the older age group who were categorized as depressed (*p* ≤ 0.001).

Table 1 presents the association between sociodemographic characteristics by depression status and by country. Table 1 also illustrates significant differences between depressed and non-depressed people for age, marital status, education, and income. Most noteworthy for the current study, findings reveal a significant association between depressed and non-depressed people for SES across all countries.

**Table 1:** Demographic characteristics of the COURAGE study sample, stratified by depression status, and by country

	Finland (%)			Poland (%)			Spain (%)		
	Depressed (*n* = 80)	Non-Depressed (*n* = 1854)	p	Depressed (*n* = 214)	Non-Depressed (*n* = 3726)	p	Depressed (*n* = 510)	Non-Depressed (*n* = 4073)	p
Sex									
Male	21.1	49.2	≤0.001	36.2	48.0	0.054	31.0	51.1	≤ 0.001
Age Group			0.21			0.04			≤0.0001
18–39	41.8	32.1		26.7	42.0		21.8	38.2	
40–64	42.1	45.2		50.0	40.9		50.1	41.9	
65+	16.1	22.7		23.3	17.1		28.1	19.9	
Marital Status			≤ 0.001			0.004			≤ 0.001
Single	31	25		23	17		21	28	
Married/Cohabiting	38	60		49	65		45	58	
Divorced/Widowed	31	15		28	13		34	14	
No. Yrs Education									
mean ± SD	12.09 ± 3.8	12.2 ± 4.1	0.819	11.05 ± 3.9	11.75 ± 3.7	0.009	8.27 ± 5.7	10.9 ± 5.6	≤ 0.001
Income Quintiles			≤ 0.001			0.004			≤ 0.001
1 (Poorest)	25.2	22.6		38.2	22.9		12.4	23.5	
2 (Poorer)	31.3	15.2		18.1	13.7		32.5	14.7	
3 (Middle)	19.3	18.1		11.1	13.8		24.2	19.6	
4 (Rich)	14.9	27.9		10.8	22.3		18.2	23.3	
5 (Richest)	9.2	16.2		21.9	27.2		12.7	18.9	
SES Index									
(0–110)	31.9 ± 22.0	38.7 ± 25.5	≤ 0.02	27.4 ± 23.6	34.9 ± 24.2	≤ 0.001	23.3 ± 21.5	32.6 ± 26.2	≤ 0.001

Logistic regression analyses were conducted to assess the association between indices of SES (education, Model 1; and income, Model 2) and the index for SES (Model 3), with depression as the outcome (see Table 2). After adjusting for the various confounders (sex, age, and marital status) the logistic regression models illustrated that for all countries (Finland, Poland and Spain), the odds of depression were significantly decreased for every unit increase in the SES index (OR 0.99, 95 % CI 0.98–0.99; OR 0.98, 95 % CI = 0.975–0.99, OR 0.99, 95 % CI 0.984–0.99, respectively). Income was found to be a significant predictor of depression in Finland and Poland, but not in Spain.

**Table 2:** Results from the multivariable logistic regression analysis on the association between indices of socio-economic status on depression by country

		Model 1		Model 2		Model 3	
		OR	95 % CI	OR	95 % CI	OR	95 % CI
Depression Finland	Yrs of Education	0.94	0.89–0.985				
	Income (Quintile)			0.84	0.714–0.987		
	SES Index (0–110)					0.991	0.98–0.997
Depression Poland	Yrs of Education	0.934	0.88–0.983				
	Income (Quintile)			0.84	0.71–0.98		
	SES Index (0–110)					0.986	0.975–0.99
Depression Spain	Yrs of Education	0.913	0.887–0.939				
	Income (Quintile)			1.0	0.914–1.09		
	SES Index (0–110)					0.989	0.984–0.995

Model 1 includes years educated, and was adjusted for sex, age, and marital status. Model 2 includes income quintiles, and was also adjusted for sex, age and marital status. Model 3 includes SES index, and was also adjusted for sex, age and marital status

Concerning the correlation of SES components by country analysis, a positive correlation was observed between education and income in Finland (rho = 0.45, *p* < 0.01), Poland (rho = 0.31, *p* < 0.01) and Spain (rho = 0.17, *p* < 0.01).

## Discussion

Our findings illustrate that for each country, higher education and a higher SES index score act as protective factors against depression. A higher income was associated with lower odds of having depression in Finland and Poland, but not in Spain. To the best of our knowledge, this is the first multi-national European study that has evaluated the association between SES as a concrete index and depression, using nationally-representative data from three countries using standardized instruments. This allowed for a comparison between the different settings (Spain, Finland and Poland), which has not been done previously.

The findings from the current study, which illustrates the impact that a higher SES has on preventing depression, reinforces findings from previous studies. Results from a meta-analysis on socioeconomic inequalities in depression conducted by Lorant and colleagues [29] revealed conclusively that low SES individuals had higher odds of being depressed. This study also found that for each additional year of education, the odds of being depressed decreased by 3 % and a 1 % increase in the income ranking led to a 0.74 decrease in the log odds of being depressed [29]. Additionally, the correlation between income and education which was found across all countries in the current study support previous studies of European regions which found that high levels of educational attainment were found to be significantly and robustly associated with higher income [37].

The findings which outline that education has a role in the prevalence of depression supports previous findings from an epidemiological study of major depressive disorder (MDD), which illustrated an inverse association between the prevalence of MDD and level of education [38]. Additionally, a large prospective study demonstrated that lower education was associated with an increased risk of depression at follow-up [20]. In the European context, a Norwegian cross-sectional study on adults found that low education levels ware significantly associated with depression [39]. This association was consistent across all the countries in the current study. In terms of income, our findings demonstrated that higher income quintiles were associated with depression, however only in Finland and Poland. Other European studies also found that personal economy was strongly and independently associated with depression [40, 41].

Additionally, our results support the use of the SES index as a composite score for SES, using both years in education and income level [32–36]. These two indicators are supported as components for the SES index as they have a strong theoretical association with depression, and the data was easily obtained and applicable to our study sample [25–29]. The multiple-indicator approach to establish SES has the advantage of providing more information and greater flexibility [33–36]. The current findings contribute to existing research in depression, illustrating that depression and low SES are inextricably linked, but it also builds upon previous research by having demonstrated that a composite score of SES based on two components of SES (education and income) can in fact predict depression symptomatology reliably across three countries.

An explanation for why higher income quintiles were not associated with depression in Spain can be postulated when considering the context of the countries at the time of data collection. At the time of the data collection, the prevalence of depression in Spain (9 %) was found to be more than double the rate of depression compared to both Finland (4 %) and Poland (4 %). This discrepancy in rates of depression can be attributed to a number of factors. Firstly, at the time of data collection, Spain was embroiled in a financial crisis, where unemployment was at a record high, exceeding 20 % [42]. For this same period, the unemployment rate in Poland and Finland were both under 10 % [43, 44]. In light of the fact that there were no methodological differences between the countries in the study design, instruments or definitions used, the discrepancy in the prevalence of depression may also be attributed to cultural differences. Such cultural variances may involve the willingness to report, the differences in the amount of stigma attached to depression between the countries (which would influence the reliability of the self-report measure due to self-presentation biases), and different environmental stressors (e.g. urbanization) [45–47]. Further research on understanding the cross-cultural differences in depression is needed in order to examine how these factors interact with each other and influence the prevalence rates.

By reason of the culture explanation, this may also be a valid explanation as to why a lower income was not found to be associated with depression in Spain. Perhaps that for Spanish people, income is not the main protective factor against depression, and the likes of good social networks [48], sunlight [49] and diet [50] may all be important protective factors against depression in Spain. The interpretation of the current evidence is complex, however, and the findings that a lower SES is associated with depression can be interpreted differently in each of the settings, as the effect of SES on depression may not be due to the same reason in all settings. For instance, living conditions, lifestyle choices, health and welfare characteristics and culture may contribute to the variability in the prevalence of depression. For example, Spain has limited mental health coverage compared to most other European countries, and therefore may account for the higher rates of depression compared to Poland and Finland. One explanation for the finding that lower income was not associated with depression in Spain may be that because 80 % of depression patients in Spain live with their families. This is a larger percentage than any other EU country [51], and may account for why household income alone is not associated with depression in Spain. In Finland, studies have found that depression is associated with retirement, which may be due to the fact that retirees are economically inactive [52] – this supports previous studies which found that household income is a significant determinant of depressive symptoms [41, 53]. Additionally, Finland has one of the strongest income gradients in health compared to other Scandinavian countries [54], and as such, it seems intuitive why in the current study, income was found to be associated with depression in Finland. Regarding interpreting the findings of the Polish data, similar circumstances can account for the finding that income is inversely associated with depression. The level of health care funding in Poland represents one of the factors which contributes to the emergence and persistence of inequalities in health, including that of depression [55], and this is reflected in the data which illustrates that income is negatively associated with depression in Poland.

### Strengths and limitations

The strength and novelty of the current study is that it is the first paper that presents the relationship between SES and depression among three European countries by applying standardized designs and procedures across all survey populations. This common methodology allows to us to investigate the role or SES in countries with different cultural and economic statuses in Europe, maximizes cross-national transferability and comparability of the findings, and serves as the first time an accurate comparison can be made in a European context. Another major strength of this paper is the large sample size that was available, which was drawn from representative samples of non-clinical populations. Moreover, regarding the nature of the study, relatively few studies have examined SES cross-nationally, not to mention focused on specific European countries that represent different cultural and economic statuses. Additionally, our research proposes a composite score for SES based on income and education, which is also novel for the outcome of depression across European countries.

Some methodological limitations should be taken in to consideration when interpreting the findings. Firstly, the cross-sectional nature of the study renders it difficult to draw any clear conclusions regarding the direction of the SES - depression relationship, and thus limits the applicability to determine whether any of the SES indices predicts depression over time. Another challenge of the current study is that there is no formal consensus regarding the definition or measurement for SES, therefore limiting comparison to past papers. Additionally, information on social security, labor market attachment and ageing outcomes were beyond the scope of the current study. Finally, the variables ‘occupation’ and ‘prestige’ were omitted from the generated SES index, which some may argue is a major shortcoming, as occupation has been historically and theoretically regarded as crucial components of SES.

## Conclusions

The SES index (composed of education and income) appears to predict depression symptomatology across European countries. In all countries, years of education but not income level was related to depression. In light of the dearth of cross-national research looking at the role of SES on depression in Europe, these findings have been enlightening; however, longitudinal studies are needed to provide further transparency regarding the direction of causality in the relationship between SES and depression. In terms of implications for policy, a concrete and valid index of SES is required in order to inform policy and research initiatives. The findings also support the notion that resources should be allocated to developing strategies to enhance economic growth and educational programmes in low SES areas in order to have positive benefits that will protect against the development and persistence of depression.

## Abbreviations

CAPI: Computer-assisted personal interviewing
CI: Confidence interval
CIDI: Composite international diagnostic interview
COURAGE: Collaborative Research on Ageing in Europe
DSM-IV: Diagnostic Statistical Manual IV
MDD: Major depressive disorder
SES: Socio-economic status
WHO: World Health Organization

## References

[1] World Health Organisation. Factsheet on Depression. World Health Organisation. 2012. http://www.who.int/mediacentre/factsheets/fs369/en/. Accessed 15 Aug 2015

[2] Lépine JP, Briley M (2011). The increasing burden of depression. Neuropsychiatr Dis Treat.

[3] Wittchen HU, Jacobi F (2005). Size and burden of mental disorders in Europe—a critical review and appraisal of 27 studies. Eur Neuropsychopharm.

[4] Kessler RC, Bromet EJ (2013). The epidemiology of depression across cultures. Annu Rev Public Health.

[5] Ayuso-Mateos JL, Vazquez-Barquero JL, Dowrick C, Lehtinen V, Dalgard OS, Casey P, Wilkinson C, Lasa L, Page H, Dunn G, Wilkinson G, ODIN Group (2001). Depressive disorders in Europe: prevalence figures from the ODIN study. Brit J Psychiatr.

[6] World Health Organization. The global burden of disease: 2004 Update. Geneva: The World Health Organization; 2008.

[7] Kovess-Masféty V, Alonso J, de Graaf R, Demyttenaere K (2005). A European approach to rural–urban differences in mental health: the ESEMeD 2000 comparative study. Can J Psychiatry.

[8] Kessler RC, Aguilar-Gxiola S, Alonso J, Chatterji S, Lee S, Ormel J, Üstün TB, Wang PS (2009). The global burden of mental disorders: an update from the WHO World Mental Health (WMH) surveys. Epidemiol Psichiatr Soc.

[9] Pikhartova J, Chandola T, Kubinova R, Bobak M, Nicholson A, Pikhart H (2009). Neighbourhood socioeconomic indicators and depressive symptoms in the Czech Republic: a population based study. Int J Publ Health.

[10] Krieger N, Williams DR, Moss NE (1997). Measuring social class in US public health research: concepts, methodologies, and guidelines. Annu Rev Publ Health.

[11] Andrade L, Caraveo-Anduaga JJ, Berglund P (2000). Crossnational comparisons of the prevalences and correlates of mental disorders. Bull World Health Organ.

[12] Muntaner C, Eaton WW, Miech R, O’Campo P (2004). Socioeconomic position and major mental disorders. Epidemiol Rev.

[13] Jo S-J, Yim HW, Bang MH, Lee MO, Jun T-Y, Choi J-S (2011). The association between economic status and depressive symptoms: an individual and community level approach. Psychiatry Investigation.

[14] Lorant V, Croux C, Weich S, Deliege D, Mackenbach J, Ansseau M (2007). Depression and socio-economic risk factors: 7-year longitudinal population study. Brit J Psychiat.

[15] Patal V, Araya R, de Lima M, Ludermir A, Todd C (1999). Women, poverty and common mental disorders in four restructuring societies. Soc Sci Med..

[16] Li Z, Page A, Martin G, Taylor R (2011). Attributable risk of psychiatric and socio-economic factors for suicide from individual-level, population-based studies: a systematic review. Soc Sci Med.

[17] Ustun T, Chatterji S, Mechbal A, Murray C, WHS Collaborating Groups (2005). Quality assurance in surveys: standards, guidelines and procedures. Household sample surveys in developing and transition countries. New York.

[18] Galobardes B, Shaw M, Lawlow DA, Lynch JW, Davy Smith G (2006). Indicators of socioeconomic position (part 1). J Epidemiol Commun H.

[19] OECD (2011). Divided we stand: why income inequality keeps rising.

[20] Eikemo TA, Huisman M, Bambra C, Kunst AE (2008). Health inequalities according to educational level in different welfare regimes: a comparison of 23 European countries. Sociol Health Illn.

[21] World Health Organization (2013). World Health Organization translation guidelines.

[22] Perales J, Martin S, Ayuso-Mateos JL (2014). Factors associated with active aging in Finland, Poland, and Spain. Int Psychogeriatr.

[23] Haro JM, Arbabzadeh-Bouchez S, Brugha TS, de Girolamo G, Guyer ME, Jin R, Lepine JP, Mazzi F, Reneses B, Vilagut G, Sampson NA, Kessler RC (2006). Concordance of the Composite International Diagnostic Interview Version 3.0 (CIDI 3.0) with standardized clinical assessments in the WHO World Mental Health surveys. Int J Methods Psychiatr Res.

[24] American Psychiatric Association (1994). Diagnostic and statistical manual of mental disorders.

[25] Kaplan GA, Roberts R, Camacho TC, Coyne JC (1987). Psychosocial predictors of depression. Prospective evidence from the human population laboratory studies. Am J Epidemiol.

[26] Sargeant K, Bruce ML, Florio LP (1990). Factors associated with 1-year outcome of major depression in the community. Arch Gen Psychiat.

[27] Miech RA, Shanahan MJ (2000). Socioeconomic status and depression over the life course. J H Soc Behav.

[28] Mirowsky J, Ross CE (1998). Education, personal control, lifestyle and health–a human capital hypothesis. Res Aging.

[29] Lorant V, Deliege D, Eaton W, Robert A, Philippot P, Ansseau M (2003). Socioeconomic inequalities in depression: a meta-analysis. Am J Epidemiol.

[30] Virtanen M, Kivimaki M, Elovainio M, Linna A, Pentti J, Vahtera J (2007). Neighbourhood socioeconomic status, health and working conditions of school teachers. J Epidemiol Commun H.

[31] Liberatos P, Link BG, Kelsey JL (1988). The measurement of social class in epidemiology. Epidemiol Rev.

[32] Katsarou A, Tyrovolas S, Psaltopoulou T, Zeimbekis A, Tsakountakis N, Bountziouka V, Gotsis E, Metallinos G, Polychronopoulos E, Lionis C, Panagiotakos D (2010). Socio-economic status, place of residence and dietary habits among the elderly: the Mediterranean islands study. Public Health Nutr.

[33] Haug MR (1977). Measurement in social stratification. Annu Rev Sociol.

[34] Stevens G, Cho JH (1985). Socioeconomic indexes and the new 1980 census occupational classification scheme. Soc Sci Res.

[35] Stravraky KM, Kincade JE, Steward MA, Donner AP (1987). The effect of socioeconomic factors on the early prognosis of cancer. J Chronic Dis.

[36] Stockwell EG (1963). A critical examination of the relationship between socioeconomic status and mortality. Am J Pub H.

[37] Rodríguez-Pose A, Tselios V (2008). Education and income inequality in the regions of the European union SERC.

[38] Gan Z, Li Y, Xie D, Shao C, Yang F, Shen Y (2012). The impact of educational status on the clinical features of major depressive disorder among Chinese women. J Affect Disord.

[39] Bjelland I, Krokstad S, Mykletun A, Dahl A, Tell GS, Tambs K (2008). Does a higher education level protect against anxiety and depression? The HUNT study. Soc Sci Med.

[40] Molarius A, Berglund K, Eriksson C, Eriksson HG, Lindén-Boström M, Nordström E, Persson C, Sahlqvist L, Starrin B, Ydreborg B (2009). Mental health symptoms in relation to socio-economic conditions and lifestyle factors–a population-based study in Sweden. BMC Public Health.

[41] Lahelma E, Laaksone M, Marikainen P, Rahkonena O, Sarlio-Lähteenkorva S (2006). Multiple measures of socioeconomic circumstances and common mental disorders. Soc Sci Med.

[42] Eurostat. EuroStat Database. Unemployment rate in Spain. European Central Bank. Brussels: European Commission. http://sdw.ecb.europa.eu/quickview.do?SERIES_KEY=132.STS.M.ES.S.UNEH.RTT000.4.000. Accessed 23 Oct 2015

[43] Eurostat. EuroStat Database. Unemployment rate in Poland. European Central Bank. Brussels: European Commission. http://sdw.ecb.europa.eu/quickview.do?SERIES_KEY=132.STS.M.PL.S.UNEH.RTT000.4.000. Accessed 23 Oct 2015

[44] Eurostat. EuroStat Database. Unemployment rate in Finland. European Central Bank. Brussels: European Commission. http://sdw.ecb.europa.eu/quickview.do?SERIES_KEY=132.STS.M.FI.S.UNEH.RTT000.4.000

[45] McKenzie K, Murray A, Booth T (2013). Do urban environments increase the risk of anxiety, depression and psychosis? An epidemiological study. J Affect Disorders.

[46] Georg Hsu LK, Wan YM, Chang H, Summergrad P, Tsang BY, Chen H (2008). Stigma of depression is more severe in Chinese Americans than Caucasian Americans. Psychiatry.

[47] Berger JL, Addis ME, Reilly ED, Green JD (2012). Effects of gender, diagnostic labels, and causal theories on willingness to report symptoms of depression. J Soc Clin Psych.

[48] Tuesca-Molina R, Fierro Herrera N, Molinares Sosa A, Oviedo Martínez F, Polo Arjona Y, Polo Cueto J, Sierra Manrique I (2003). Socializing groups as protective factor against depression in elderly people. Barranquilla, Colombia.

[49] Kent ST, McClure LA, Crosson WL, Arnett DK, Waley VG, Sathiakumar N (2009). Effect of sunlight exposure on cognitive function among depressed and non-depressed participants: a REGARDS cross-sectional study. Environ Health.

[50] Skarupski KA, Tangney CC, Li H, Evans DA, Morris MC (2013). Mediterranean diet and depressive symptoms among older adults over time. J Nutr Health Aging.

[51] Gomez-Beneyto M, Asensio A, Berenguer MJ, Espinosa J (1996). Desinstitucionalización de enfermos mentales crónicos sin recursoscomunitarios. Cronicicidad en Psiquiatria.

[52] Wiggins RD, Schofield P, Sacker A, Head J, Bartley M (2004). Social position and minor psychiatric morbidity over time in the British household panel survey 1991–1998. J Epidemiol Commun H.

[53] Meertens V, Scheepers P, Tax B (2003). Depressive symptoms in the Netherlands 1975–1996: a theoretical framework and an empirical analysis of socio-demographic characteristics, gender differences and changes over time. Sociol Health Illn.

[54] Huijts T, Eikemo TA, Skalicka V (2010). Income-related health inequalities in the Nordic countries: examining the role of education, occupational class, and age. Soc Sci Med.

[55] World Health Organization (2012). Social inequalities in health in Poland.

